# Counting Finger and Wrist Movements Using Only a Wrist-Worn, Inertial Measurement Unit: Toward Practical Wearable Sensing for Hand-Related Healthcare Applications

**DOI:** 10.3390/s23125690

**Published:** 2023-06-18

**Authors:** Shusuke Okita, Roman Yakunin, Jathin Korrapati, Mina Ibrahim, Diogo Schwerz de Lucena, Vicky Chan, David J. Reinkensmeyer

**Affiliations:** 1 Department of Mechanical and Aerospace Engineering, University of California Irvine, Irvine, CA 92697, USA; okitas@uci.edu; 2 Department of Anatomy and Neurobiology, University of California Irvine, Irvine, CA 92697, USA; 3 College of Computing, Georgia Institute of Technology, Atlanta, GA 30332, USA; romario.yakunin@gmail.com; 4 Department of Electrical Engineering and Computer Science, University of California Berkeley, Berkeley, CA 94720, USA; jkorr@berkeley.edu; 5 Department of Biomedical Engineering, University of California Irvine, Irvine, CA 92697, USA; minai1@uci.edu; 6 AE Studio, Venice, CA 90291, USA; diogo@ae.studio; 7 CAPES Foundation, Ministry of Education of Brazil, Brasilia 70040-020, Brazil; 8 Rehabilitation Services, University of California Irvine, Irvine, CA 92697, USA; vchan2@hs.uci.edu

**Keywords:** human activity recognition (HAR), neural network, rehabilitation, convolutional neural network (CNN), the inertial measurement unit (IMU), motion capture system (MC), stroke, wearable sensing

## Abstract

The ability to count finger and wrist movements throughout the day with a nonobtrusive, wearable sensor could be useful for hand-related healthcare applications, including rehabilitation after a stroke, carpal tunnel syndrome, or hand surgery. Previous approaches have required the user to wear a ring with an embedded magnet or inertial measurement unit (IMU). Here, we demonstrate that it is possible to identify the occurrence of finger and wrist flexion/extension movements based on vibrations detected by a wrist-worn IMU. We developed an approach we call “Hand Activity Recognition through using a Convolutional neural network with Spectrograms” (HARCS) that trains a CNN based on the velocity/acceleration spectrograms that finger/wrist movements create. We validated HARCS with the wrist-worn IMU recordings obtained from twenty stroke survivors during their daily life, where the occurrence of finger/wrist movements was labeled using a previously validated algorithm called HAND using magnetic sensing. The daily number of finger/wrist movements identified by HARCS had a strong positive correlation to the daily number identified by HAND (R^2^ = 0.76, *p* < 0.001). HARCS was also 75% accurate when we labeled the finger/wrist movements performed by unimpaired participants using optical motion capture. Overall, the ringless sensing of finger/wrist movement occurrence is feasible, although real-world applications may require further accuracy improvements.

## 1. Introduction

The hand plays a critical role in daily functions. A wide variety of conditions, including trauma to the hand, developmental disorders such as Autism spectrum disorder [1], and neurologic injuries such as stroke [2] and spinal cord injury [3], diminish effective hand use. The overuse of the hand can also cause pain and injury, such as carpal tunnel syndrome [4]. The treatment for these conditions usually relies at least in part on achieving targeted levels of daily hand activity, with the goal of promoting recovery by gradually increasing use or avoiding injury by limiting use. However, currently, there are a few non-obtrusive wearable sensors for quantifying daily hand use. This limits the ability of clinicians and patients to understand if target hand-use amounts are being met and to adapt treatment plans.

Currently, there are several promising wearable approaches to finger movement sensing, including: (1) wearing a camera, typically around the neck, and inferring hand activity using computer visions [5]; (2) wearing a ring with an inertial measurement unit (IMU) and inferring hand activity from the motion of the ring [6]; and (3) wearing a magnetic ring and inferring hand activity based on the changes in magnetic fields sensed at the wrist [2,7,8,9]. We recently used this last approach to confirm the hypothesis that real-world upper extremity (UE) hand use increases only for stroke survivors who achieve a threshold level of UE functional capability [9]. Here, we explored an even less intrusive approach that was suitable for implementation with the hardware available in many commercial activity trackers that are worn similarly to watches.

Our working hypothesis was that we could use machine learning to identify the vibrational patterns produced at the distal end of the forearm by active flexion and extension of the fingers and wrist. Previous studies have shown that vibrations induced by tapping the forearm can be read out using sensors in an armband, highlighting the fact that there is informational content in vibrations that propagate through the forearm [10]. Here, we studied, for the first time to our knowledge, whether finger/wrist movements produced vibrations at the wrist that could be used to identify the occurrence of finger/wrist movements. To this end, we proposed a novel approach for hand activity recognition using a convolutional neural network with spectrograms, named Hand Activity Recognition through a Convolutional neural network with Spectrograms (HARCS).

## 2. Methods

### 2.1. Wrist-Worn Sensors

We used data from two wrist-worn sensors in this study: the Manumeter and the MiGo. Both devices are non-commercial devices developed in collaboration with the company Flint Rehabilitation Devices, LLC (Irvine, CA, USA).

The Manumeter (see Figure S1 in Supplementary Materials) is a watch-like device with inertial and magnetic sensing capabilities, the latter of which we have studied extensively in several previous studies [2,7,8,9]. For this study, we made use of the six degrees of freedom (DOF) Inertial Measurement Unit (LSM6DSL) it incorporates; the range of the accelerometer was set to ±4G, and the gyroscope was set to ±500 degrees per second, both with a 16-bit resolution. The Manumeter was equipped with an ARM Cortex M4 CPU (NRF52, Nordic Semiconductor, Trondheim, Norway) and a real-time clock (PFC2123) to calculate the time and the date. It had a 4G flash memory (MT29F4G01ADAGDWB-IT:G TR) that recorded data obtained from the IMU. The sampling rate was set as 52.6 Hz, which is high enough for capturing human movements [11]. The Manumeter had an OLED display that could show information, such as the time of day or the number of hand movements performed by a wearer. In previous studies, for counting finger/wrist movements, the user also wore a magnetic ring on the index finger. A magnetic array in the wrist unit sensed changes in the magnetic field produced by the ring (see [9]).

The MiGo is also a watch-like device with IMU sensing, but it does not have magnetic sensing. It uses the same IMU (LSM6DSL), but the accelerometer range was set to ±2 G, and the gyroscope range was set to ±500 degrees per second, both with a 16-bit resolution). It contains the same real-time clock and microcontroller as the Manumeter. An integrated 2.4 gHz radio is used to stream data from the IMU. IMU data are read and pre-processed at 205 Hz, then streamed at 100 Hz using the enhanced shock-burst wireless protocol. We down-sampled IMU data to 52.6 Hz offline using Scipy’s resample function [12]. The MiGo also has an OLED display and a push-button available and is powered by a 90 mAh LiPo battery.

To summarize, for all the experiments, we used the same IMU with the same sampling rate, and this was mostly achieved with the Manumeter. We only used the MiGo for seven subjects in the Mocap-Lab Dataset, as described in more detail below.

### 2.2. Experiments and Data Sets

For this project, we needed to obtain IMU recordings taken during UE movements that sometimes included finger/wrist movements, and we also needed to know when a finger/wrist movement occurred in order to train the network and then validate its capability. We relied on three datasets, which we named the dataset based on: (1) the way we identified whether a finger/wrist movement occurred (i.e., via the Manumeter or motion capture); and (2) the location of collection (at home or in the laboratory). All experiments were approved by the UCI Institutional Review Board, and subjects provided informed consent.

*Manumeter-Home Dataset*: For this dataset, we used IMU recordings from 20 people with a stroke (16 male, 4 female) who wore the Manumeter at home, which was acquired as part of a previously reported clinical trial on the feasibility and efficacy of hand count feedback [2]. We labeled movements using the HAND algorithm, which was previously developed in our laboratory [9]. The HAND algorithm recognizes hand movements based on fluctuations in the magnetic field produced from the magnetic ring worn on the finger, detecting if field changes are over the pre-set thresholds [13]. This algorithm identifies finger flexion-extension movements with an accuracy of around 85%. Further details can be found in [9].

*Manumeter-Lab Dataset*: We also acquired IMU recordings in a laboratory-based experiment from the same subjects with a stroke who participated in generating the Manumeter-Home Dataset. These subjects performed an exercise where they moved their hand or wrist a fixed number of times at a fixed pace by following a video prompt [2]. We also recruited an additional 7 unimpaired subjects to participate in a similar experiment, except these subjects performed an exercise where they moved their arm while keeping their hand still. Similar to the Manumeter-Home Dataset, we labeled finger/wrist movements using the HAND algorithm [9].

**Figure 1:**
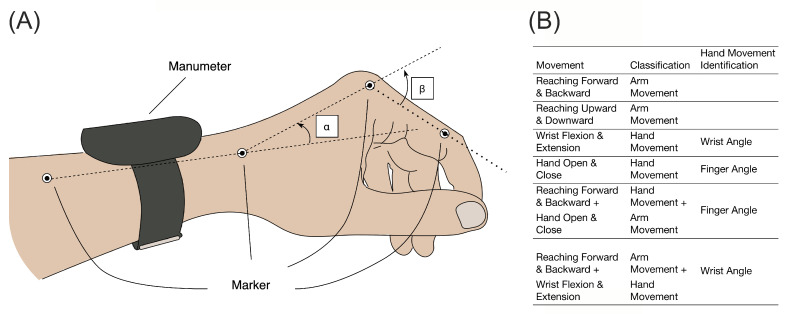
(**A**) Marker placements for inferring the wrist and finger angle. α represents the wrist angle. Additionally, β represents the finger angle (i.e., metacarpophalangeal joint angle). Four markers were taped as shown in the figure to the wrist and finger to obtain α and β. (**B**) The list of movements in the Mocap-Lab Dataset. Subjects performed 6 movements involving an arm-only movement, hand-only movement, and hand and arm movement.

*Mocap-Lab Dataset*: To further validate the HARCS algorithm, we also acquired IMU recordings from nine unimpaired male volunteers who performed a series of structured UE movements while wearing either the Manumeter or the MiGo in a laboratory. For this experiment, we used an optical motion capture system (Phasespace, San Leandro, CA, USA, nine cameras) to measure wrist and finger angles (Figure 1A). The participants performed six movements that involved various combinations of hand, wrist, and arm movements (Figure 1B). The subjects performed the same movement continuously over a 90 s period (at a self-selected rate, typically around 0.5–1 Hz), then rested for 15 s before performing the next movement in the order shown in Figure 1B. We manually counted how many movements each subject performed. The dataset annotation process involved down-sampling Mocap data from 480 Hz to 52.6 Hz to match the IMU data and calculating finger and wrist angles. Instantaneous angular rates were computed by differentiating these angles, and a window was labeled positive if a peak was found (see below for how windows were defined). Thresholds were set to match the number of peaks with the number of hand movements.

### 2.3. Data Preprocessing

**Figure 2:**
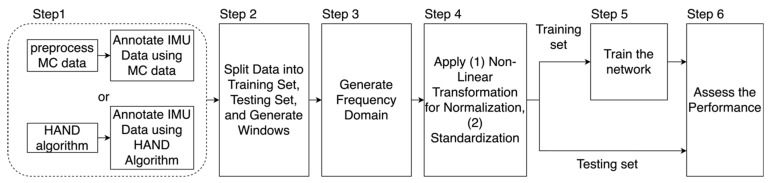
The 6 steps for training and assessing the neural network.

**Figure 3:**
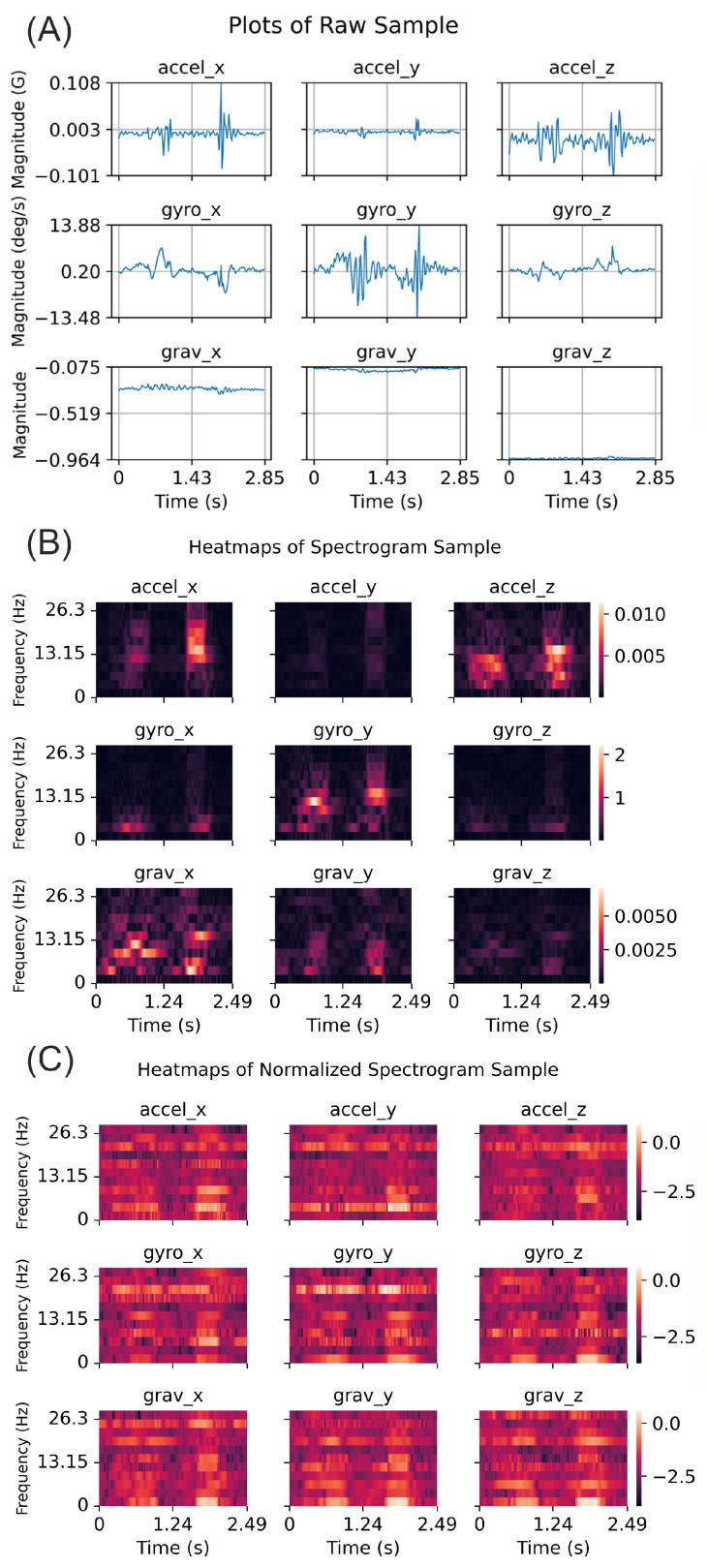
An example of processing a data-sample where the HAND algorithm predicted the existence of a hand movement. This data-sample contained 150 time-samples from the IMU. (**A**) Acceleration, gyro, and gravity vector measures are shown in each row as a function of time, with the columns indicating the *x*, *y*, and *z* axes of the sensor. (**B**) The heatmaps of the spectrograms computed from this sample for each measurement. (**C**) The heatmaps of the normalized spectrograms.

We used 6 steps to annotate and preprocess the datasets and to train the network (Figure 2). We used 9 features in training the network: 3-axis acceleration, 3-axis angular velocity, and the 3-axis gravity direction. Note that we subtracted gravity from the acceleration and obtained gravity direction through the Madgwick filter [14]. In Step 1, we annotated these datasets using either the motion capture system or the HAND algorithm. In Step 2, we split data into training and testing sets (see details in the Section 2.5). Then, we generated windows with 150 samples (i.e., about 2.85 s of data), which served as inputs for the training and validation datasets. For the training set, these windows were generated with a stride of 50-time samples (about 0.95 s of data). We labeled a window as positive if at least a one-time sample was designated as positive for a hand movement within the last 50-time samples of that window. We adopted this approach to provide the network with an adequate context to determine if a hand movement had taken place, i.e., the network evaluated a window of 150-time samples to predict if a hand movement took place in the last 50-time sample in that window. As a result, for every 50-time samples, we assigned a label of either positive or negative for hand movement occurrence and defined these 50-time samples, as well as the previous 100-time samples, as the “data-sample” for performing identification (see Figure 3 for an example of a data-sample). In Step 3, we converted the samples of raw sensor signals for each data sample into spectrograms by applying a Short-Time Fourier Transform (STFT). In Step 4, we applied a non-linear transformation and the Box–Cox transform [15,16,17,18]) to the spectrogram variables for normalization, which we found to improve network performance. During the generation of the training set, the parameter λ for the Box–Cox transform was selected using a SciPy scalar optimizer [12]. After this normalization, we applied standardization by subtracting out the mean and dividing by the standard deviation. Further details can be found in the Supplementary Materials.

### 2.4. Convolutional Neural Network Design

**Figure 4:**
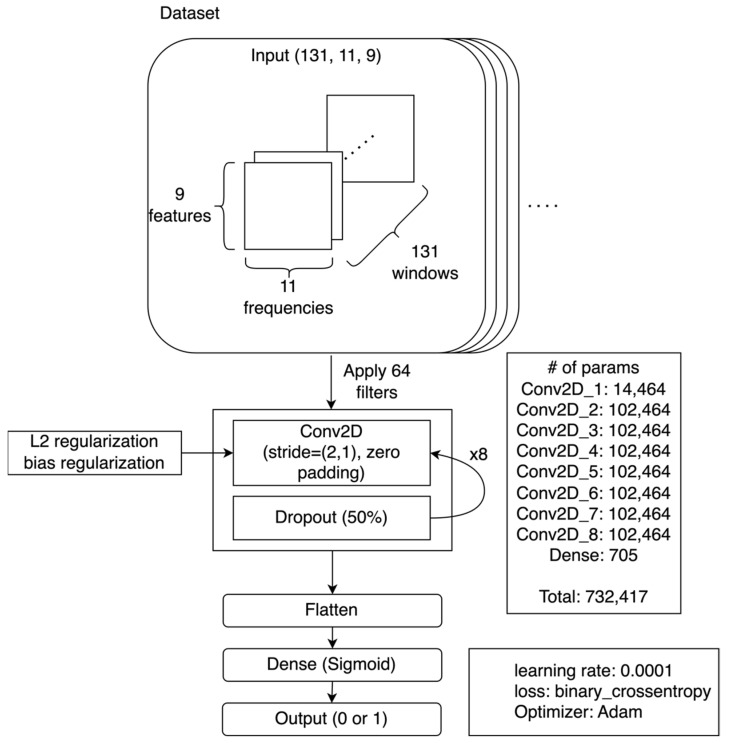
An example of the CNN architecture trained by the Manumeter-Home Dataset.

*In Step 5*, we trained a Convolutional Neural Network (CNN): a network architecture often used in image recognition tasks (Figure 4) [19]. Spectrogram classification can be effectively executed with CNNs [20], and there are various studies available on the application of spectrograms for classification tasks, specifically within the realms of voice [21] and human activity recognition [22]. The CNN we used consisted of 7–8 repeated convolutional layers with L2 regularization and dropout layers. The dropout layers were inserted to avoid overfitting [23]. A sigmoid activation function was used to output the final predicted probabilities. For the loss function, we used a binary cross-entropy loss function with an Adam optimizer [24].

Each data sample was formatted as 131 × 9 × 11, which is a transformed version of the original 150-time samples data with 9 sensor variables. The Short-Time Fourier Transform (STFT) was applied using a window size of 20, shifting one-time sample at a time. This analysis produced 131 windows from the initial 150-time samples.

Tensorflow and Keras 2.0 were used to implement the CNN [25,26]. We manually tuned the following parameters depending on a network: (1) the number of convolution layers; (2) the convolution kernel size; (3) the number of convolution filters; (4) whether norm layers were present; (5) l2 regularization lambda. We trained the network using either the Mocap-Lab Dataset or Manumeter-Home Dataset. A summary of the parameters in each case is provided in the Supplementary Materials.

### 2.5. Training the Network

We trained CNN with different IMU datasets, which, to review, were taken in different settings and with finger/wrist movements identified in different ways. We asked the following questions:

*Q1. How well can we identify in-the-wild hand movements made by people with varying levels of hand impairment due to stroke?*

Our largest dataset was the one obtained across a day during home activities by people with a stroke, i.e., the Manumeter-Home Dataset. We asked: can a CNN trained on IMU recordings and obtained from the daily use of UE at home by people with a stroke recognize when finger/wrist movements occur? To answer this question, we used leave-one category-out-cross-validation (LOOCV) based on subjects’ impairment levels. We trained HARCS by splitting the data into training and testing sets according to the subjects’ Fugl-Meyer Upper Extremity (UEFM) scores. The UEFM score is a clinical scale ranging from 0 (severe impairment) to a maximum of 66 (no impairment), assessing motor function and joint functioning in individuals with post-stroke [27]. For instance, we trained HARCS using subjects’ data in a 30 ≤ UEFM score < 66 when we assessed subjects’ data in the range of a UEFM score < 30. We labeled the occurrence of a finger/wrist movement using the previously validated HAND algorithm implemented with the magnetic sensing capability of the Manumeter, which showed about 80% accuracy [9]. In addition to the LOOCV method based on subjects’ impairment levels, we also implemented an alternative approach to evaluate the performance of the CNN, which we called the random 5-fold cross-validation grouped by participants. In this method, we randomly divided the dataset, allocating 80% of the subjects for training and 20% for testing without taking into account the subjects’ UEFM scores. This approach aimed to assess the robustness and generalizability of our CNN model when mixing a diverse range of hand impairments due to stroke into the training data. We performed 6 iterations of this random fold process, each time creating a new training and testing data split and measuring the accuracy of the classification.

The accuracy estimates depended on the accuracy of the HAND algorithm used to label this data set. Previously, through extensive laboratory testing, we found the accuracy of the HAND algorithm to be about 85% [9]. To estimate the bounds on the true accuracy of HARCS, we used Bayes’ theorem:

(1) $$\begin{array}{cc} P(HARCS_{correct})= & P(HARCS_{correct}|HAND_{correct})\;\times \;P(HAND_{correct})\\ & +P(HARCS_{correct}|HAND_{incorrect})\;\times \;P(HAND_{incorrect}) \end{array}$$

where $P(HARCS_{correct})$ is the true accuracy of HARCS; $P(HARCS_{correct}|HAND_{correct})$ is the accuracy of HARCS given that the HAND algorithm is correct (which ranges between 74% and 81%; see Section 3); and $P(HAND_{correct})$ is the accuracy of the HAND algorithm (85%). Finally, $P(HARCS_{correct}|HAND_{incorrect})\;$ is the accuracy of the HARCS algorithm when the HAND algorithm is incorrect. We did not know this last probability; therefore, we let it vary from 0 to 1 in the analysis presented in Section 3 below to find bounds on the true accuracy of HARCS.

We also compared HARCS to three other machine learning approaches: the support vector machine (SVM), k-nearest neighbor (kNN), and a multi-layer perceptron, following the approaches by [28,29], and implementing the algorithms in scikit-learn [30]. See the Supplemental Materials for the parameter selection of the other approaches.

We also studied how the identification accuracy depended on finger-wrist movement speed. To conduct this, we calculated the mean of the acceleration amplitude across the 150 data points in each window. After we created a validation set, we split the data with respect to the amplitude of movements to compute a weighted confusion matrix in each range to average the effect of skewness. We averaged this based on the number of actual positives and actual negatives.

*Q2. Is HARCS Sensitive to Isolated Hand Movements?*

A second question we asked was: Can a CNN, trained using the Manumeter-Home Dataset, accurately count hand movements when a person performs structured movements comprised hand-only movements and, conversely, not count movements comprised arm-only movements? To answer these questions, we used the Manumeter-Lab Dataset. Since hand and arm movements often occur together in the “wild”, our goal here was to understand the unique sensitivity of the algorithm to isolated hand movements and, conversely, its susceptibility to arm-only movements.

*Q3. Does HARCS Identify Structured Hand Movements with Accurate Labeling?*

Using the Manumeter to label movements introduced an error because the HAND algorithm was about 85% accurate [9]. Therefore, we asked: Can a CNN trained on movements counted accurately using motion capture recognize when a finger/wrist movement has occurred? We also varied the type of movement (hand only, arm only, and hand arm together) to study the accuracy of these movement types. To answer these questions, we trained and evaluated CNN using the Mocap-Lab Dataset with LOOCV. We first removed one subject’s data as the validation data set and then trained the CNN on the data from the remaining eight subjects. We repeated the same process 9 times, using each subject’s data for validation. We used the testing set to evaluate how well the CNN detected the presence or absence of finger/wrist movements for each of the six movements shown in Figure 1B. Moreover, for the Mocap-Lab Dataset, we implemented two distinct training approaches to evaluate the potential impact of different labeling methods on the network’s performance. In the first approach, we only labeled hand-only movements as positives; that is, we labeled combined hand/arm movements as negative. This tested how well the network could identify when hand-only movements occurred in isolation from arm movements. In the second approach, we labeled combined hand/arm movements as positives. This tested how well the network could identify when hand movements occurred with or without arm movement.

### 2.6. Statistical Analysis and Performance Analysis

To characterize the network performance, we used *Accuracy*, *Precision*, *Recall*, and the *F*1-*Score*, as defined in Equations (2)–(5), which are all widely used metrics [31], Additionally, we generated receiver operating characteristic (ROC) curves and associated Area Under the Curve (AUC) values to assess the performance of the model’s binary predictions [32]. We also computed a Pearson correlation to compare hand counts between HARCS and the HAND algorithm.

(2) $$Precision=\frac{True\;Positive}{True\;Positive+False\;Positive}$$

(3) $$Recall=\frac{True\;Positive}{True\;Positive+False\;Negative}$$

(4) $$F1\;score=2\;\times \;\frac{\;Precision\;\times \;Recall\;}{\;Precision+Recall\;}$$

(5) $$Accuracy=\frac{True\;Positive+True\;Negative}{True\;Positive+False\;Positive+False\;Negative+True\;Negative}\;\times \;100$$

## 3. Results

We tested the ability of HARCS to identify finger/wrist movements from wrist-worn IMU data sets obtained at home from people with hand impairments after a stroke and in the lab from both stroke and unimpaired subjects. HARCS uses CNN to recognize finger/wrist movement occurrence based on the velocity/acceleration spectrograms these movements create.

### 3.1. HARCS Can Identify Unstructured Hand Movements in People with Stroke in-the-Wild across a Wide Range of Impairment Levels

**Figure 5:**
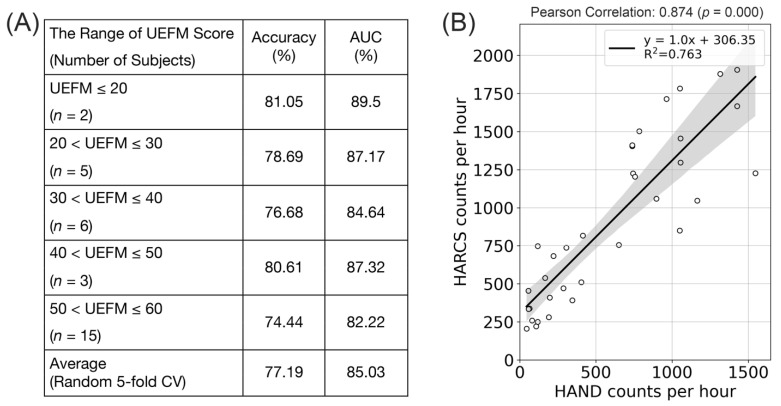
(**A**) Accuracies and AUCs for HARCS-based identification of finger/wrist movements in-the-wild for 20 stroke subjects with varying UEFM scores. (**B**) The correlation between HARCS and HAND counts of finger/wrist movement occurrence, where HAND counts were produced in a previous study using information from a magnetic ring.

We divided the 20 stroke subjects into groups according to a standard clinical measure of UE movement ability: the UEFM Score. For reference, a UEFM score of less than 20 means severe UE paresis, a score of 30–40 means that a hand function is just emerging; and a score of 66 means normal movement ability [33]. HARCS accuracy was 81% for the two subjects in the range of 0–20 for the UEFM score and 74% for 15 subjects in the range of 50–60; that is, the accuracy varied by 7% for the level of hand impairment (Figure 5A). Across all participants, using a random 5-fold CV, the average accuracy was 77%. Further, the number of HARCS counts identified across a day ’s worth of wear time was strongly correlated with the number of HAND counts, which were the counts identified in the previous study that used additional information from a magnetic ring instead of just IMU data (r = 0.874, *p* = 0.00, R^2^ = 0.763) (Figure 5B).

These accuracy numbers should be taken with a caveat: they depend on the accuracy of the HAND algorithm, which we found previously through extensive laboratory testing to be about 85% [9]. To estimate the bounds on the true accuracy of HARCS, we used Bayes’ theorem, as described in the Section 2. If the average accuracy of HARCS in the matching HAND was 77%, then the true accuracy of HAND (to identify the true presence or absence of a hand movement) was between 65 and 80%. The exact value depended on how well HARCS identified a hand movement accurately when HAND did not—a currently unknown parameter.

Using the same Manumeter-Home Dataset, we compared four machine-learning approaches (see Supplementary Materials for details). The various measures of network performance we examined were highest for HARCS, including the R^2^ value for the regression between HARCS and HAND counts (Table 1).

**Figure 6:**
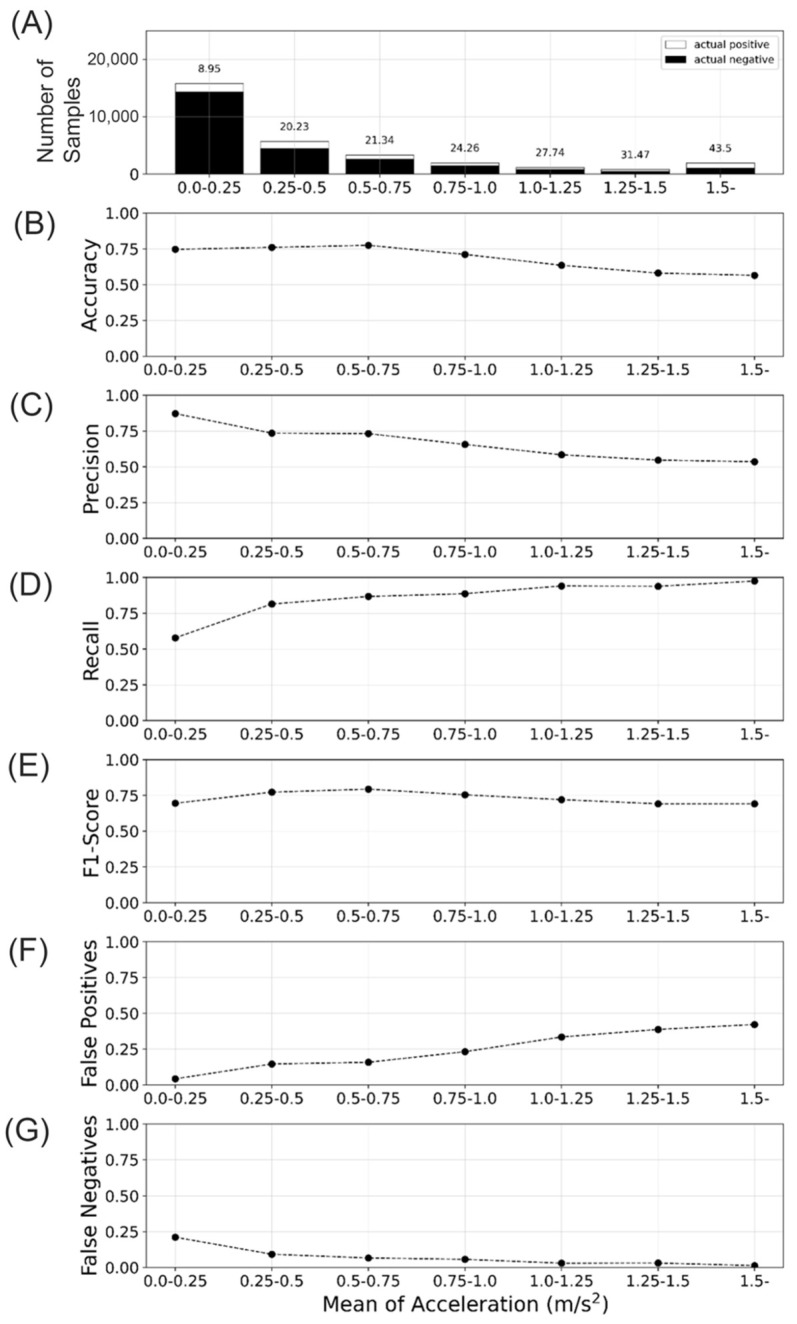
The relationship between the network performance and the mean linear acceleration across the data-sample window of duration ~2.8 s. (**A**) Number of data samples segmented by mean acceleration. (**B**–**E**) Performance metric segmented by the mean acceleration. (**B**) Accuracy. (**C**) Precision, (**D**) Recall. (**E**) F1-score. (**F**,**G**) Proportion of classifications segmented by the mean acceleration. (**F**) False positive rate. (**G**). False negative rate. In (**A**), the proportions of actual positives and total data-samples are shown on the top of each bar.

Again, using the same Manumeter-Home Dataset, we analyzed how the HARCS classification performance depended on the average speed of limb movements in the data-sample window (Figure 6). The number of samples decreased with the increasing average speed (Figure 6A). Accuracy and precision decreased with an increasing average speed, while Recall increased (Figure 6B–D). The F1 score stayed consistent with increasing speed (Figure 6E). The decrease in precision could be attributed to an increase in false positives as speed increased (Figure 6F), while the increase in recall was due to a reduction in false negatives as the speed increased (Figure 6G). Despite the balanced metrics, this outcome indicated that the network had a tendency to predict positive outcomes when the mean of accelerations was high. In other words, as the acceleration increased, the network was more likely to identify a movement as positive, leading to a higher rate of false positives and consequently lowering Precision. Conversely, a decrease in false negatives contributed to the increase in Recall, demonstrating that the network more effectively-recognized actual positive cases.

### 3.2. HARCS Sensitivity to Hand-Only and Arm-Only Movement

**Figure 7:**
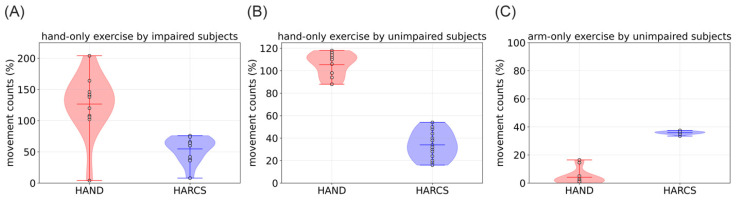
Comparison of HARC algorithm counts versus HAND counts and the true number of hand movements for (**A**) Hand-only exercises by people with a stroke (**B**) Hand-only exercises by unimpaired subjects, (**C**) Arm-only exercise by unimpaired subjects. For (**A**,**B**), perfect counting resulted in 100% movement counts. For (**C**), perfect counting resulted in 0% movement counts, since C was arm-only exercise. A value of 100% represents 50 hand movements for the hand-only exercise and 200 movements for the arm-only exercise.

After we trained HARCS on the Manumeter-Home Dataset, we tested its abilities on the data set from impaired and unimpaired subjects in which subjects performed hand-only or arm-only movements. This was of interest because hand and arm movements often occur naturally together in the wild, and we wanted to understand how sensitive HARCS is to isolated hand movement. HARCS was able to count hand-only movements but underestimated the actual number of hand movements performed by both impaired and unimpaired subjects by 45% and 66%, respectively. This performance was less accurate than the HAND algorithm, which overestimated the counts by 26 and 5%, respectively (Figure 7A,B). For the arm-only exercise, HARCS should not have counted any hand movements but counted 36% of arm movements as also corresponding to a hand movement: a false-positive rate that was again larger than HAND (Figure 7C).

### 3.3. HARCS Can Identify Structured Hand Movements, but Accuracy Depends on Movement Type

**Figure 8:**
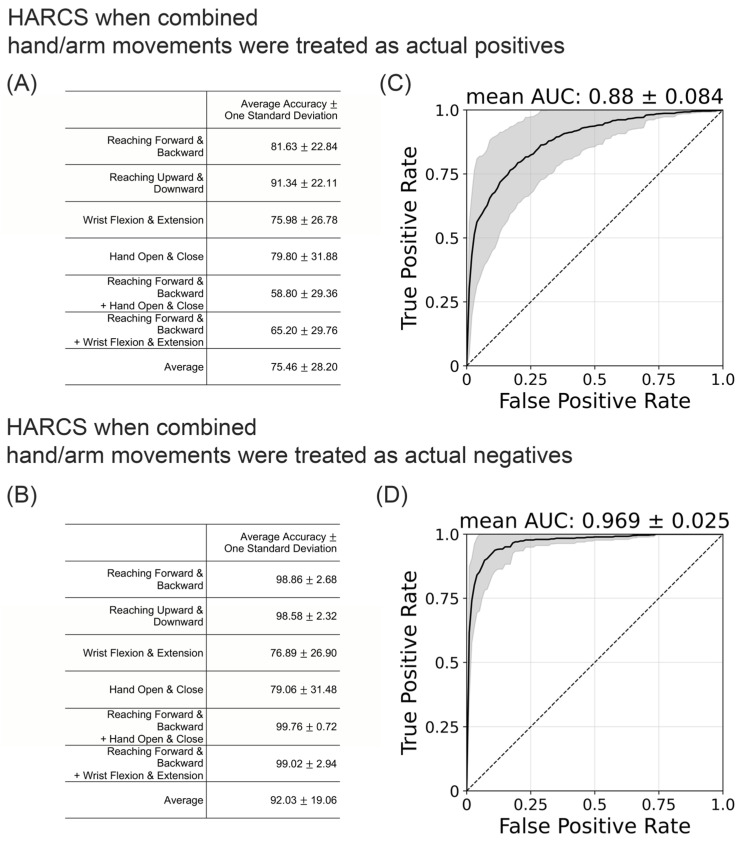
(**A**,**B**) Average accuracy of HARCS when trained and tested on the Mocap-Lab Dataset obtained from nine unimpaired subjects. (**C**,**D**) The mean of the ROC Curve (solid line) in which the shaded area represents ± 1 standard deviation. In (**A**,**C**), combined hand/arm movements were treated as an actual positive. In (**B**,**D**), combined hand/arm movements were treated as an actual negative.

As a further test of the core idea of identifying hand movements from wrist IMU recordings, we performed a laboratory experiment that generated the Mocap-Lab Dataset. For this experiment, subjects repeatedly made different types of movements involving the hand and/or arm (Figure 1B). Hand movement occurrence was identified based on an optical motion capture system, removing any labeling error that resulted from depending on the HAND algorithm for labeling. The average accuracy for HARCS in the Mocap-Lab Dataset when combined hand/arm movements were treated as actual positives was 75%, but this accuracy varied depending on the type of movement that the subject performed (Figure 8A). HARCS performed well in identifying isolated finger/wrist movements (80 and 76% accuracy, see Figure 8A) and in ignoring the vibrations created by arm movements only (81 and 91% accuracy). HARCS struggled to accurately identify finger/wrist movements when combined with arm/hand movements (59 and 65% accuracy). Across all movement types, the average AUC was 0.88 (Figure 8C). By contrast, the average accuracy for HARCS for the Mocap-Lab Dataset when combined with hand/arm movements and treated as negative was 92% on average, showing the ability of the network to more accurately identify hand-only movements (Figure 8B,D).

## 4. Discussion

Our goal in this study was to determine if we could identify the occurrence of hand movements using only a wrist-worn IMU. The algorithm that we designed, called HARCS, focuses on recognizing the vibrational patterns produced by the flexion and extension of the fingers and wrist. Using a dataset obtained from twenty stroke survivors during their daily life, the daily number of finger/wrist movements identified by HARCS had a strong positive correlation with the number identified by a previously developed hand-movement recognition algorithm that used a magnetic ring and magnetometer (HAND). This was true across a wide range of hand impairment levels after stroke. Further experiments in the lab found that HARCS had an accuracy of ~75% and was sensitive to the occurrence of hand-only movements, although it tended to undercount hand-only movements. These results demonstrate the feasibility of the ringless sensing of hand movement using wrist-worn IMUs. In the following, we discuss the performance of HARCS as well as its limitations and directions for future research.

### 4.1. Discussion of HARCS Performance

In contrast to existing approaches to hand activity detection [9,34], one of the advantages of HARCS is it does not require any other components or devices except a wrist-worn IMU. Thus, HARCS could potentially be deployed immediately with a wide range of existing smartwatches. Encouragingly, the hand counts produced by HARCS were strongly correlated with the HAND algorithm, which required additional hardware, including a magnetic ring and magnetometer array. This strong correlation was present even though the HAND algorithm is known to be susceptible to false positives due to stray magnetic fields in the environment [9], introducing noise into the labeling.

Two key design features of HARCS were to convert IMU data to the frequency domain before using it in classification and to use a CNN network, an architecture that has previously been found useful for image recognition tasks [19]. Essentially, HARCS can be viewed as performing image recognition on the nine spectrograms corresponding to the nine types of features produced by finger, wrist, and arm movements. It is unclear which of the features are most important for performance, and this is also an important direction for future research.

In the first laboratory experiment, we found that HARCS was sensitive to isolated hand movements but undercounted it by ~30%. Further, HARCS assigned false positives at a rate of about 40% to arm-only movements. In this experiment, we trained the network using the in-the-wild data from the stroke subjects, which may account for some inaccuracies. As mentioned above, we labeled the in-the-wild data with the HAND algorithm, which was imperfect. Further, the movement types performed in the lab were only a small subset of the ones the network was trained on (i.e., daily life movements), and this may have biased the network in a suboptimal way for the laboratory tests. Finally, the testing data for arm-only movement were from unimpaired subjects, while the training data were from stroke subjects. Stroke subjects tend to move more slowly, and we found that the network was sensitive to the average movement speed in the data window. Future work should more closely explore the effect of movement speed since different patient populations may move at different speeds. It may be possible to improve HARCS’ performance for specific applications by better matching training data to the application. For example, speed effects might be addressable by training networks for groups of people at different impairment levels.

In the second laboratory experiment that used the motion capture system to accurately label data, we found that network performance was excellent when we trained the network to identify when hand-only movement occurred. Network performance decreased when we trained the network to identify when any type of hand movement occurred, including those occurring simultaneously with an arm movement. This highlighted that there was a confounding nature to the vibrations produced by arm movements when trying to identify hand movements, as was also evident in the algorithm producing false positives for arm-only movements. This confound may present a fundamental limit to the accuracy possible with HARCS, although this remains an open question. Future work should explore which vibrational patterns are unique to hand-only, hand-plus-arm, and arm-only movements to gain a deeper insight.

### 4.2. Limitations and Future Directions

A key limitation of the current work is that the in-the-wild data analysis relied on the magnetometer-based HAND algorithm for labeling, which we know from previous studies has some inaccuracies. We calculated the theoretical bounds for how HAND inaccuracy can affect HARCS accuracy but obtaining a more accurate ground truth is desirable. It may be possible to improve performance by generating a more accurately labeled in-the-wild dataset with a more obtrusive method, such as wearing instrumented gloves [7], wearable cameras [5,35], or stretchable e-textile sensors [36,37].

Other limitations include the limited sample size, including a predominance of male participants, and testing only people with a stroke. Testing HARCS with a larger and more diverse population is necessary to generalize results. IMUs are known to have drift and noise and too may have affected accuracy.

It is unclear if the accuracy of HARCS is high enough to enhance rehabilitation outcomes for people with a stroke. Poor accuracy could demotivate patients. We are not aware of studies that have defined the lower limits of accuracy required for wearable devices to motivate individuals. Commercial step trackers, which in aggregate have been proven useful [38], vary in accuracy from 70–90% [39,40], and HARCS is at the lower end of this range. An important direction for future research is to understand how the accuracy of a wearable device impacts the motivation or other desired outcomes of a hand-movement sensor. Our previous study involving the Manumeter, which had an accuracy of about 85%, demonstrated that subjects increased their daily frequency of hand movements in response to the display feedback from the device [2].

It is also unclear how big the clinical need will be for identifying hand movements only without arm movements in daily life once appropriate technology becomes clinically available. For focal injuries to the hand, such as arising from repetitive stress injuries, it seems important to define the movement “dose” that the hand receives, to prevent injury and promote recovery. Similarly, in as much as hand recovery occurs independently of arm recovery after neurologic injuries such as stroke, it seems desirable to measure the “dose” of rehabilitation that the hand itself receives. We have shown previously using the Manumeter that, while arm use is a strong predictor of hand use, the slope of the relationship varies by up to a factor of ~12, depending on the task being performed [41]. Thus, analyzing the relative amount of hand and arm use may give more insight into UE recovery than analyzing arm use alone because the spread reflects the nature of the tasks being performed.

In future research, enhancing the performance of the current HARCS system might be accomplished through various strategies, such as using ensemble learning, regularization based on movement speeds, or data augmentation to address the imbalance between true negatives and true positives. Enhancements might also be achieved in the data processing pipeline, with data augmentation simulating different movement characteristics, potentially improving the network’s robustness and generalization capabilities. The size of the detection interval could also be optimized.

For future work, we aim to incorporate non-obtrusive hand movement sensing into home rehabilitation after a stroke, allowing for real-time feedback and the analysis of patient hand movements: an approach that already showed promise with a more cumbersome magnetic sensing approach [2]. To achieve this goal, we seek to embed HARCS into a wrist-worn sensor to provide real-time hand activity recognition, allowing us to test if the current level of HARCS accuracy is sufficient for this application.

## Figures and Tables

**Table 1:** Summary of performances by four different machine learning approaches.

	KNN	SVM	Perceptron	HARCS
Accuracy	61.82	66.87	72.35	77.19
F1 score	44.22	61.37	70.71	77.98
Precision	82.03	73.61	75.18	76.02
Recall	30.51	52.81	66.98	80.62
R^2^	0.648	0.31	0.533	0.763

## Data Availability

The data presented in this study are available on request from the corresponding author. The data are not publicly available due to privacy.

## Supplementary Materials

The following supporting information can be downloaded at: https://www.mdpi.com/article/10.3390/s23125690/s1, Figure S1: (A) The Manumeter consists of a ring with an embedded magnet and a wrist unit containing an inertial measurement unit (IMU) and magnetometers. (B) The four magnetometers are placed at the corners of the wrist unit, and the IMU is positioned along the edge of the board next to the screen. Taken from [9]. Figure S2: (Left) An illustration of data processing for a single subject using the Short-Time Fourier Transform (STFT). The STFT creates a (9 features × 11 variables) window to form a spectrogram in 150-time samples. In total, 131 windows were generated from 150 samples. Figure S3: An example of data conversion using the Box–Cox transformation. (A) The distribution of the amplitude of the spectrum for each sensor measurement. The X-axis represents the amplitude of the signal generated by 9 sensor variables at the selected frequency of 7.89 Hz (i.e., 3-axis acceleration without gravity, 3-axis angular velocity from Gyroscope, and 3-axis vector in gravity). This frequency is one of the 11 discrete frequencies that were obtained by dividing the frequency range up to the Nyquist frequency (26.3 Hz) into equal intervals. The Y-axis shows the density distribution of the value, and the integral over the X-axis became 1.0. (B) The distributions of the amplitude of the spectrum after the Box–Cox transformations. Figure S4: An example of the heat map of the chosen parameters in the Box–Cox transformation. (A) The combination of the lambdas parameters. The lambda parameters were optimally selected to make each distribution close to the normal distribution. The X-axis represents 9 features used in training, and the y-axis represents the frequencies determined by the FFT size. (B) The post-Box–Cox spectrogram means. (C) The post Box-Cox spectrogram standard deviations. Table S1: Parameter settings for proposed networks. Table S2: Model comparison with accuracy (%) for Random 5-fold CV and UEFM folds (i.e., LOOCV in the main text) based on subjects’ impairment levels. The table shows the mean accuracy for Random 5-fold CV, where the dataset was randomly partitioned into training and testing data without considering the UEFM score of the subjects (six iterations), and the mean accuracy for specific UEFM folds, where the data was split based on subjects’ impairment levels.
