# Supplementary material for: Counting Finger and Wrist Movements Using Only a Wrist-Worn, Inertial Measurement Unit: Toward Practical Wearable Sensing for Hand-Related Healthcare Applications
Source: Sensors (Basel). 2023 Jun 18;23(12):5690. doi: 10.3390/s23125690 (PMC10300978; doi:10.3390/s23125690)
Supplement: Supplementary file 1 [file sensors-23-05690-s001.zip › sensors-2382773-supplementary.pdf]

sensors

MDPI

Supplementary Materials

Counting Finger and Wrist Movements Using Only a Wrist-Worn, Inertial Measurement Unit: Toward Practical Wearable Sensing for Hand-Related Healthcare Applications

Shusuke Okita [1,2], Roman Yakunin [3], Jathin Korrapati [4], Mina Ibrahim [5], Diogo Schwerz de Lucena [6,7], Vicky Chan [8] and David J. Reinkensmeyer [1,2,5,*]

[1] Department of Mechanical and Aerospace Engineering, University of California Irvine, Irvine, CA 92697, USA; okitas@uci.edu
[2] Department of Anatomy and Neurobiology, University of California Irvine, Irvine, CA 92697, USA
[3] College of Computing, Georgia Institute of Technology, Atlanta, GA 30332, USA; romario.yakunin@gmail.com
[4] Department of Electrical Engineering and Computer Science, University of California Berkeley, Berkeley, CA 94720, USA; jkorr@berkeley.edu
[5] Department of Biomedical Engineering, University of California Irvine, Irvine, CA 92697, USA; minai1@uci.edu
[6] AE Studio, Venice, CA 90291, USA; diogo@ae.studio
[7] CAPES Foundation, Ministry of Education of Brazil, Brasilia 70040-020, Brazil
[8] Rehabilitation Services, University of California Irvine, Irvine, CA 92697, USA; vchan2@hs.uci.edu
* Correspondence: dreinken@uci.edu

1. Wearable Sensors Used for This Study

This study used two wrist-worn sensors, the Manumeter (Figure S1) and the MiGo, which were developed in collaboration with Flint Rehabilitation Devices. The Manumeter features inertial and magnetic sensing with six degrees of freedom Inertial Measurement Unit (IMU), an ARM Cortex M4 CPU, and a real-time clock. The MiGo, which does not contain magnetic sensing, shares the same IMU, clock, and microcontroller as the Manumeter, with different accelerometer settings and data streaming capabilities. Both devices employed the same IMU and sampling rate, primarily using the Manumeter, with the MiGo used for only seven subjects in the Mocap-Lab Dataset.

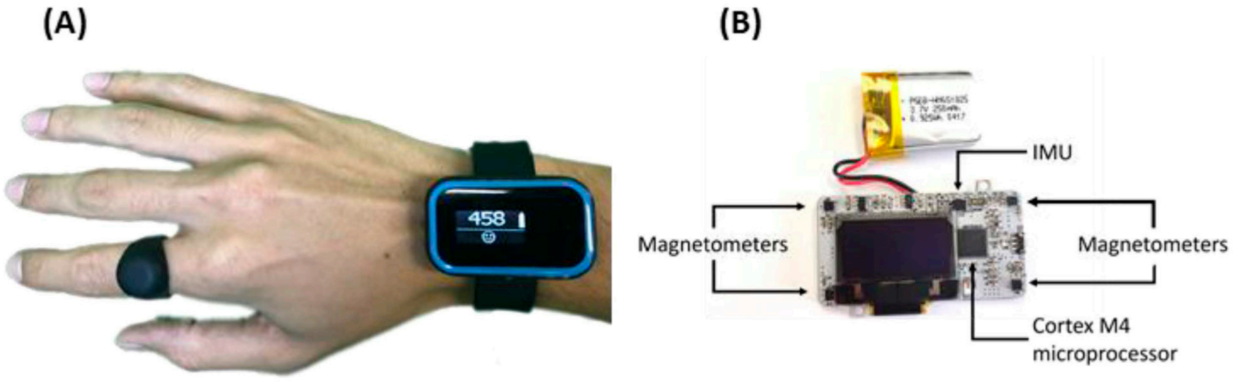

Figure S1. (A) The Manumeter consists of a ring with an embedded magnet and a wrist unit containing an inertial measurement unit (IMU) and magnetometers. **(B)** The four magnetometers are placed at the corners of the wrist unit, and the IMU is positioned along the edge of the board next to the screen. Taken from [1].

2. Inactivity Filtering

In *the Manumeter-Home Dataset* and *Manumeter-Lab Dataset*, there existed significant periods of inactivity during which all measurements remained approximately constant. The participants most likely took off the Manumeter during this time or rested with their hands on a table or their lap, suggesting it was inappropriate to assess the network's performance at these intervals. We excluded windows where the inactive regions were detected; we defined an inactive sample as a time sample during which the difference between the minimum and maximum values of all nine measurements—the x, y, and z components of the acceleration, angular velocity, and gravity direction—failed to exceed their respective thresholds, thus appearing approximately constant. The nine thresholds were calculated based on four of the data files in the *Manumeter-Home Dataset*, which were selected due to having long intervals of continuous inactivity. The largest difference in minimums and maximums was computed across the intervals in the four files for each of the nine measurements, yielding the thresholds.

3. Resampling for Imbalanced Dataset

Generally, there were significantly more negatively labeled samples than positively labeled samples, which was particularly true for the *Manumeter-Home Dataset* as participants did not perform specific movements during a pre-set amount of time. For validation sets, we under-sampled negatively labeled samples to weigh each class equally in the calculation of accuracy. We also performed this for the training sets, as otherwise, the network would be biased toward classifying a sample negatively.

4. Transformation to the Frequency Domain

We computed the spectrograms of data samples by applying a Short-Time Fourier Transform (STFT) [36]: a signal analysis technique for determining the sinusoidal frequency and the phase content of local parts of a signal. We selected 20-time samples as the FFT size and utilized a Tukey window with a shape parameter of 0.25. The FFT size for the time sampling rate 52.6Hz produced 2.63 Hz increments in generating a spectrum, resulting in the generation of 11 frequencies from 0 Hz to the Nyquist frequency 26.3 Hz. The windows had an overlap of 19-time steps in order to convey as much information as possible within the resulting spectrogram. The spectrograms were generated for each measurement type, resulting in each sample containing nine spectrograms stacked along the last axis and a sample with a shape of (131-time steps, 11 frequencies and nine measurements) (Figure S3).

$$\mathrm{STFT}\{x[n]\}(m,\omega) = X(m,\omega) = \sum_{n=-\infty}^{\infty} x[n]\omega[n-m]e^{-j\omega n} \quad (1)$$

Here, x[n] represents the signal and $\omega[n-m]$ represents the adapted Tukey window.

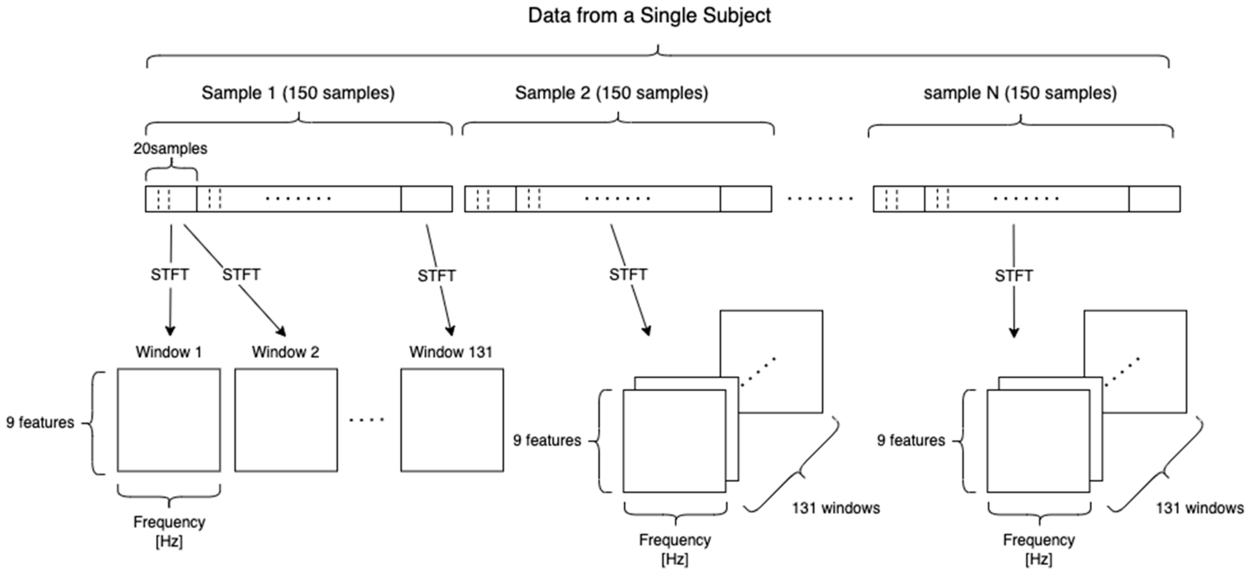

Figure S2. (Left) An illustration of data processing for a single subject using the Short-Time Fourier Transform (STFT). The STFT creates a (9 features × 11 variables) window to form a spectrogram in 150-time samples. In total, 131 windows were generated from 150 samples.

5. Non-Linear Transformation of the Spectrograms

The generated spectrograms did not have normal distributions in values and had a significantly larger density for low amplitudes. Therefore, after converting all the samples in the datasets to spectrograms, we applied a Box–Cox transformation that converted skewed distributions to normal distributions [2–5]. Equation 2 shows the formula we used to convert the time series signals:

$$x^{\lambda} = \begin{cases} \dfrac{x^{\lambda} - 1}{\lambda} & \text{if } \lambda \neq 0 \\ \log\, x & \text{if } \lambda = 0 \end{cases} \tag{2}$$

where x and λ represent the input value and the parameter varying a scaling of data distribution, respectively. λ was chosen independently for every combination of frequency and measurement type, resulting in an array of lambdas with a shape of (11 frequencies and nine measurements). During the generation of the training set, λ was chosen to maximize the log-likelihood function using the SciPy scalar optimizer [6]. Afterward, the mean and standard deviations (SDs) of the dataset were calculated along the sample and time axes, again resulting in arrays with shapes of (11 frequencies and nine measurements). The computed mean and SDs normalized the entire dataset. The same λ, means, and SDs from the training sets were used to generate the validation set.

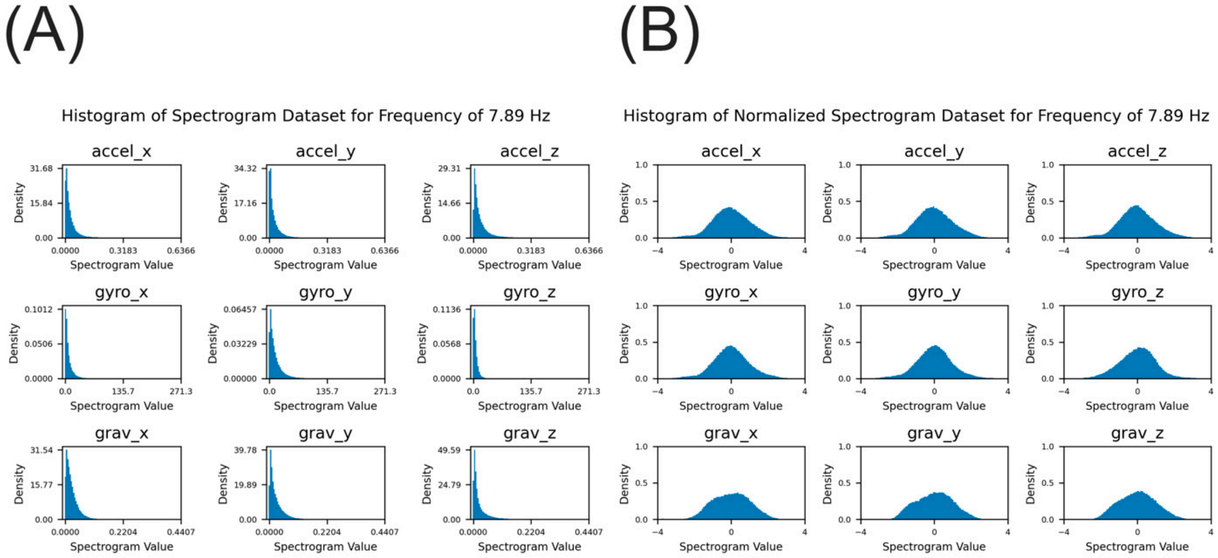

Figure S3. An example of data conversion using the Box–Cox transformation. **(A)** The distribution of the amplitude of the spectrum for each sensor measurement. The X-axis represents the amplitude of the signal generated by 9 sensor variables at the selected frequency of 7.89 Hz (I.e., 3-axis acceleration without gravity, 3-axis angular velocity from Gyroscope, and 3-axis vector in gravity). This frequency is one of the 11 discrete frequencies that were obtained by dividing the frequency range up to the Nyquist frequency (26.3 Hz) into equal intervals. The Y-axis shows the density distribution of the value, and the integral over the X-axis became 1.0. **(B)** The distributions of the amplitude of the spectrum after the Box–Cox transformations.

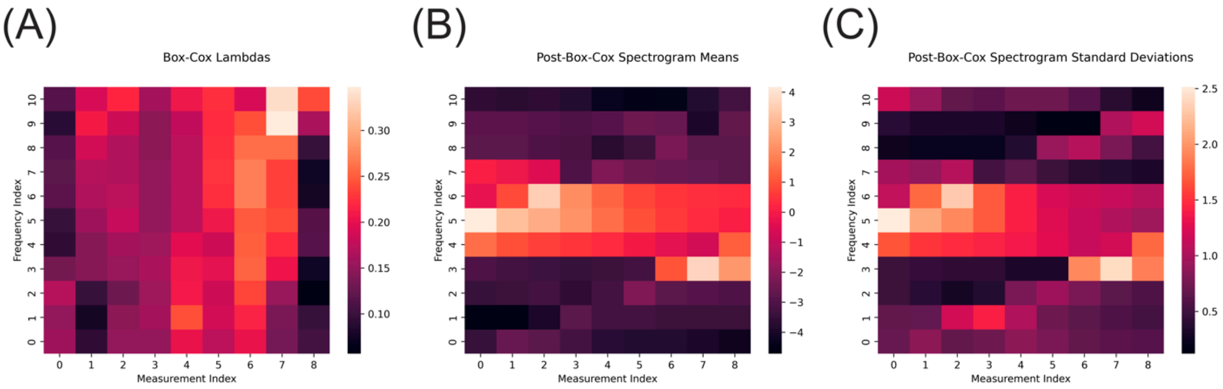

Figure S4. An example of the heat map of the chosen parameters in the Box–Cox transformation. **(A)** The combination of the lambdas parameters. The lambda parameters were optimally selected to make each distribution close to the normal distribution. The X-axis represents 9 features used in training, and the y-axis represents the frequencies determined by the FFT size. **(B)** The post-Box–Cox spectrogram means. **(C)** The post Box-Cox spectrogram standard deviations.

6. Model Comparisons

We compared the performances of CNN to three other machine learning methods: (1) K-Nearest Neighbor (KNN), (2) Support Vector Machine (SVM), and (3) Multi-Layer Perceptron. KNN classifies a given data point by finding the majority class of its k nearest neighbors, and we used the Scikit-learn implementation of the KNN. We experimentally set k as equal to 11, which yielded the best results for the KNN. An SVM attempted to linearly separate the two classes of points while maximizing the margin between them, and we used the Scikit-learn SGDClassifier implementation of SVM. To speed up the training of SVM, we applied the Scikit-learn implementation of the Nystroem

approximation with an RBF kernel to the training data, which reduced the dimension of the data to 100. The perceptron consisted of four 100-unit dense layers with the ReLU activation and a dropout rate of 0.5 applied after each layer, as well as a single-unit output layer with sigmoid activation. L2 regularization with a lambda of 0.0005 was applied to the weights and biases of the multi-layer perceptron.

Table S1 displays the selected parameters for each network trained using the Manumeter-Home dataset and the Mocap-Lab dataset. For the network trained with the Mocap-Lab dataset, we made adjustments to the parameters, such as modifying the convolutional layer size and incorporating a normalization layer alongside the convolutional and dropout layers to enhance learning stability.

Table S1. Parameter settings for proposed networks.

Data set used for training	The number of Convolutional layers and Dropout layers	The convolution kernel size	The number of filters for the first convolution layer	The normalization layer was used	L2 regularization lambda parameter
Manumeter-Home Dataset	8	(5, 5)	128	No	0.001
Mocap-Lab Dataset (Hand/Arm movement set as actual positive)	7	(3, 3)	100	Yes	0.004
Mocap-Lab Dataset (Hand/Arm movement set as actual negative)	7	(3, 3)	100	Yes	0.004

In this study, we compared the performance of four machine learning methods: K-Nearest Neighbors (KNN), Support Vector Machines (SVM), Multi-layer Perceptron, and Convolutional Neural Networks (CNN). For the KNN, SVM, and Perceptron methods, we tested their performances on (A) the raw samples, where we created one-dimensional vectors using raw sensor IMU signals, and (B) the samples after spectrogram preprocessing and Box–Cox normalization. To use these methods with spectrogram data, we reshaped the spectrograms into one-dimensional vectors by flattening the two-dimensional matrices, allowing them to be used as the input for KNN, SVM, and Perceptron. However, the proposed CNN was designed to work specifically with spectrograms, as it can provide a two-dimensional representation of the time-varying frequency content of the signals, allowing the CNNs to effectively capture local patterns. As a result, we only tested the custom CNNs on the spectrogram data. Therefore, our comparison included a total of seven combinations of machine learning models and data processing options, the accuracies of which are displayed in Table S2 for each test.

To evaluate the performance of our models, we used two different data-splitting methods. The first method called the Random 5-Fold Cross Validation (CV), grouped by participants, involved randomly partitioning the dataset into training and testing data without considering the UEFM score of the subjects. This approach aimed to assess the robustness and generalizability of our CNN model when mixing a diverse range of hand impairments due to stroke into the training data. We performed six iterations of this random fold process, each time creating a new training and testing data split. The second method, which we referred to as UEFM folds (i.e., LOOCV in the main text), involved splitting the data into training and testing data based on subjects' impairment levels, as determined by their UEFM scores. For instance, we trained HARCS using subjects' data in the range of a $30 \leq$ UEFM score < 66 when assessing subjects' data in the range of a

UEFM score < 30. This approach allowed us to evaluate the performance of the models across different impairment levels.

Table S2. Model comparison with accuracy (%) for Random 5-fold CV and UEFM folds (i.e., LOOCV in the main text) based on subjects' impairment levels. The table shows the mean accuracy for Random 5-fold CV, where the dataset was randomly partitioned into training and testing data without considering the UEFM score of the subjects (six iterations), and the mean accuracy for specific UEFM folds, where the data was split based on subjects' impairment levels.

	(A) Spectrograms not used			(B) Spectrograms used			
	**KNN**	**SVM**	**Perceptron**	**KNN**	**SVM**	**Perceptron**	**CNN**
Random 5-Fold CV (Six Iterations, Overall Mean)	61.82	66.87	72.35	73.86	74.54	76.46	77.19
UEFM Folds							
[0, 20)	61.14	62.1	70	78.38	78.1	79.81	81.05
[20, 30)	60.71	68.8	76.97	74.94	77.27	78.46	78.69
[30, 40)	58.46	65.92	71.94	69.64	73.92	76.3	76.68
[40, 50)	61.77	72.8	75.23	77.43	79.66	80.56	80.61
[50, 60)	58.99	62.36	64.31	71.97	73.38	74.59	74.44
Mean	60.21	66.4	71.69	74.47	76.47	77.94	78.29

References

1. Schwerz de Lucena, D.; Rowe, J.; Chan, V.; Reinkensmeyer, D.J. Magnetically Counting Hand Movements: Validation of a Calibration-Free Algorithm and Application to Testing the Threshold Hypothesis of Real-World Hand Use after Stroke. *Sensors* **2021**, *21*, 1502, doi:10.3390/s21041502.
2. Sakia, R.M. The Box-Cox Transformation Technique: A Review. *J. R. Stat. Soc. Ser. Stat.* **1992**, *41*, 169–178, doi:10.2307/2348250.
3. Bicego, M.; Baldo, S. Properties of the Box–Cox Transformation for Pattern Classification. *Neurocomputing* **2016**, *218*, 390–400, doi:10.1016/j.neucom.2016.08.081.
4. Cheddad, A. On Box-Cox Transformation for Image Normality and Pattern Classification. *IEEE Access* **2020**, *8*, 154975–154983, doi:10.1109/ACCESS.2020.3018874.
5. Atkinson, A.C.; Riani, M.; Corbellini, A. The Box–Cox Transformation: Review and Extensions. *Stat. Sci.* **2021**, *36*, 239–255, doi:10.1214/20-STS778.
6. SciPy 1.0: Fundamental Algorithms for Scientific Computing in Python | Nature Methods Available online: https://www.nature.com/articles/s41592-019-0686-2?report=reader (accessed on 3 April 2022).
